# Weighted gene co-expression network analysis to explain the relationship between plasma total carotenoids and lipid profile

**DOI:** 10.1186/s12263-019-0639-5

**Published:** 2019-05-08

**Authors:** Bénédicte L. Tremblay, Frédéric Guénard, Benoît Lamarche, Louis Pérusse, Marie-Claude Vohl

**Affiliations:** 1 Institute of Nutrition and Functional Foods (INAF), Laval University, 2440 Hochelaga Blvd, Quebec City, QC G1V 0A6 Canada; 2 School of Nutrition, Laval University, 2425 rue de l’Agriculture, Quebec City, QC G1V 0A6 Canada; 3 Department of Kinesiology, Laval University, 2300 rue de la Terrasse, Quebec City, QC G1V 0A6 Canada

**Keywords:** Carotenoids, French Canadians, Hub genes, Lipid profile, WGCNA

## Abstract

**Background:**

Variability in circulating carotenoids may be attributable to several factors including, among others, genetic variants and lipid profile. However, relatively few studies have considered the impact of gene expression in the inter-individual variability in circulating carotenoids. Most studies considered expression of genes individually and ignored their high degree of interconnection. Weighted gene co-expression network analysis (WGCNA) is a systems biology method used for finding gene clusters with highly correlated expression levels and for relating them to phenotypic traits. The objective of the present observational study is to examine the relationship between plasma total carotenoid concentrations and lipid profile using WGCNA.

**Results:**

Whole blood expression levels of 533 probes were associated with plasma total carotenoids. Among the four WGCNA distinct modules identified, turquoise, blue, and brown modules correlated with plasma high-density lipoprotein cholesterol (HDL-C) and total cholesterol. Probes showing a strong association with HDL-C and total cholesterol were also the most important elements of the brown and blue modules. A total of four and 29 hub genes associated with total carotenoids were potentially related to HDL-C and total cholesterol, respectively.

**Conclusions:**

Expression levels of 533 probes were associated with plasma total carotenoid concentrations. Using WGCNA, four modules and several hub genes related to lipid and carotenoid metabolism were identified. This integrative analysis provides evidence for the potential role of gene co-expression in the relationship between carotenoids and lipid concentrations. Further studies and validation of the hub genes are needed.

**Electronic supplementary material:**

The online version of this article (10.1186/s12263-019-0639-5) contains supplementary material, which is available to authorized users.

## Background

Carotenoids are a reliable biomarker of fruit and vegetable consumption [1, 2]. Over 90% of the daily carotenoid intakes are provided by fruits and vegetables [3]. Carotenoids are a family composed of more than 700 fat-soluble pigments, but α-carotene, β-carotene, β-cryptoxanthin, lutein, lycopene, and zeaxanthin represent over 95% of total circulating carotenoids in human plasma or serum [3, 4]. Inter-individual variability in circulating carotenoids has been observed and may be attributable to several factors including age, sex, body weight, physical activity, genetic, and lipid profile [2]. Accordingly, lower total cholesterol (TC), LDL-cholesterol (LDL-C), and HDL-cholesterol (HDL-C) concentrations were associated with lower circulating carotenoids [5]. Plasma HDL-C concentrations also mediated the observed sex difference in serum carotenoids in Caucasian individuals [5]. Correlations were observed between α- and β-carotene and HDL-C and triglyceride (TG) concentrations [6] and between β-cryptoxanthin and zeaxanthin and TG in a previous study by our group [7]. The NHANES 2003–2006 study also reported that several serum carotenoids positively correlated with HDL-C concentrations [8]. Thus, the link between circulating carotenoids and plasma lipid profile has been observed in several studies and is plausible considering that the majority of circulating carotenoids are associated with lipoproteins [9].

Carotenoid intakes are inversely associated with the risk of cardiovascular diseases and certain cancers [10]. They may mediate their effects mainly via antioxidant properties but also via other mechanisms such as gap junction communication, cell growth regulation, immune response, and modulation of gene expression [11, 12]. Several genome-wide association studies have identified genetic variants that influence circulating carotenoid concentrations [13–16]. Genetic variants may cause differences in the absorption, assimilation, distribution, metabolism, and excretion of carotenoids [13, 17, 18]. Carotenoids have also been shown to regulate the expression of genes protective against carcinogenesis and inflammation [19]. Carotenoids and their derivatives (e.g., retinoid) may exert their effect on gene expression via several transcriptional systems such as the retinoid receptors, the nuclear factor-kappa B, and the peroxisome proliferator-activated receptors [20]. However, relatively few studies have considered the impact of gene expression in the inter-individual variability in circulating carotenoid concentrations. Gene expression may represent a potential mechanism that links carotenoids to lipid profile. In addition, no previous study has examined the interconnection between plasma carotenoids, lipid profile, and genome-wide gene expression levels. Most studies considered expression of genes individually and ignored the high degree of interconnection between genes. Weighted gene co-expression network analysis (WGCNA) is a widely used systems biology approach used to identify gene clusters (modules) with highly correlated expression levels, to relate the modules to phenotypic traits, and to identify key hub genes within modules that are related to the phenotypic traits [21].

Thus, the objective of the present observational study is to examine the relationship between plasma total carotenoid concentrations and lipid profile using WGCNA. The hypothesis was that genome-wide gene expression levels are associated with plasma total carotenoids and that clusters of genes associated with carotenoids are correlated to lipid profile. To test this hypothesis, genes associated with total carotenoids were identified using regressions and WGCNA was used to identify specific modules and key hub genes related to lipid profile.

## Results

### Characteristics of study participants

Characteristics and biochemical parameters of participants are presented in Table 1. Fathers and mothers had significant differences in HDL-C concentrations. Concentrations of all six main carotenoids (α-carotene, β-carotene, β-cryptoxanthin, lutein, lycopene, and zeaxanthin) and total carotenoids measured in the fasting state are presented in Additional file 1: Table S1.

**Table 1:** Characteristic and biochemical parameters of study subjects

Biochemical parameters	Fathers (*n* = 6)	Mothers (*n* = 16)	Boys (*n* = 18)	Girls (*n* = 8)
Age (years)	42.0 ± 2.8	42.4 ± 6.0	11.9 ± 3.8	10.1 ± 2.0
BMI (kg/m^2^)	24.8 ± 1.3	23.5 ± 3.4	–	–
BMI percentile	–	–	48.9 ± 32.7	52.1 ± 29.8
TC (mmol/L)	4.70 ± 0.61	4.67 ± 0.55	4.35 ± 0.53	4.15 ± 0.48
HDL-C (mmol/L)^1^	1.36 ± 0.33	1.73 ± 0.35	1.56 ± 0.28	1.54 ± 0.31
LDL-C (mmol/L)	2.83 ± 0.67	2.53 ± 0.49	2.36 ± 0.42	2.20 ± 0.52
ApoB100 (g/L)	0.89 ± 0.22	0.77 ± 0.11	0.72 ± 0.12	0.67 ± 0.15
TG (mmol/L)	1.13 ± 0.35	0.88 ± 0.34	0.94 ± 0.39	0.89 ± 0.41
Total carotenoids (μmol/L)	5.77 ± 2.93	6.57 ± 2.23	5.88 ± 2.37	5.30 ± 1.09

All values are means ± SD

*ApoB100* apolipoprotein B-100, *BMI* body mass index, *HDL-C* high-density lipoprotein cholesterol, *LDL-C* low-density lipoprotein cholesterol, *SD* standard deviation, *TC* total cholesterol, *TG* triglycerides

^1^Means are significantly different (*p* ≤ 0.05) between fathers and mothers

### Associations between total carotenoids and genome-wide gene expression levels

Normalized gene expression levels of 18,160 probes were tested for associations with plasma total carotenoid concentrations. A total of 533 probes were associated (*P* ≤ 0.05) with total carotenoids (Additional file 2: Table S2). WGCNA was then conducted on this subset of probes, which were associated with total carotenoids in order to put them in relationship with lipid profile.

### Weighted gene co-expression network analysis (WGCNA)

**Fig. 1:**
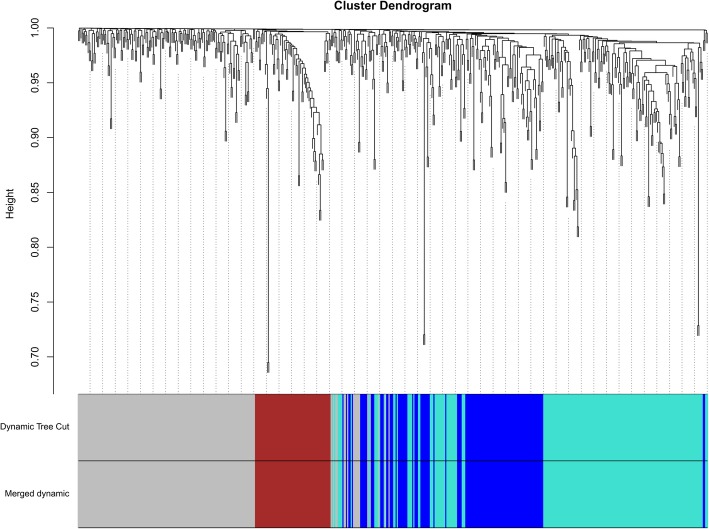
Gene dendogram obtained using average linkage hierarchical clustering. Four module colors are shown correspondingly. The merged dynamic yielded same modules as the dynamic tree cut using a cutoff of 0.25

**Fig. 2:**
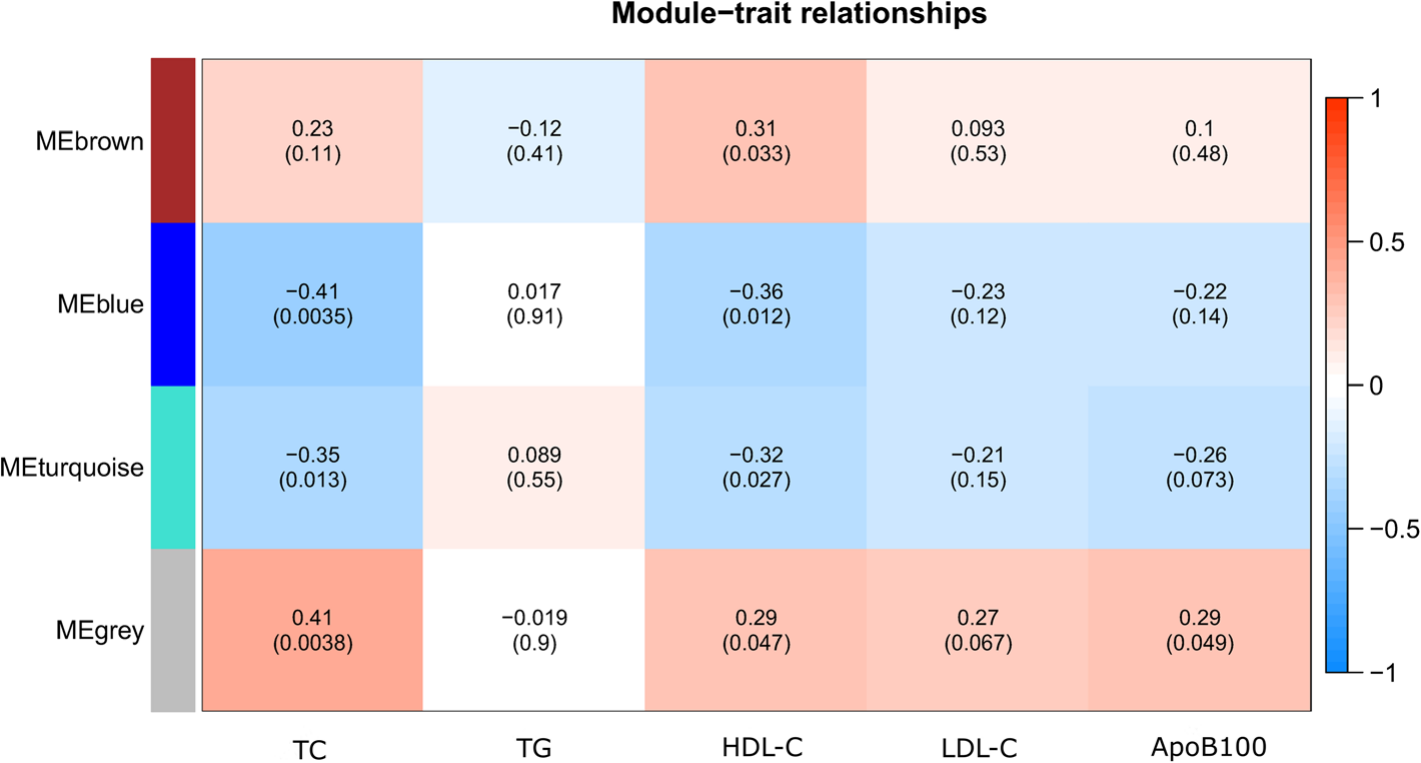
Heatmap of module-trait relationships depicting correlations between module eigengenes and lipid profile traits. Numbers in the table correspond to the correlation *r* and the *p* value in parentheses. The degree of correlation is illustrated with the color legend

A total of four distinct modules were identified from gene expression levels of the 533 probes using a dynamic tree cutting algorithm (Fig. 1). The blue, brown, turquoise, and gray modules contained 117, 64, 184, and 168 genes, respectively. The 168 uncorrelated genes were assigned to the gray module, which was excluded from further analysis. Using a cutoff height of 0.25 for the clustering of module eigengenes (MEs), none of the four modules merged. Thus, the merged dynamic yielded same modules as the dynamic tree cut (Fig. 1). To find modules of interest, correlations between MEs and lipid profile traits (TC, LDL, HDL, TG, apolipoprotein B-100 (ApoB100)) were computed. According to the heatmap of module-trait correlations (Fig. 2), ME of the turquoise module (184 genes) correlated inversely with HDL-C (*r* = − 0.32, *p* = 0.03) and TC (*r* = − 0.35, *p* = 0.01), while ME of the blue module (117 genes) correlated inversely with HDL-C (*r* = − 0.36, *p* = 0.01) and TC (*r* = − 0.41, *p* = 0.004), and ME of the brown module (64 genes) correlated positively with HDL-C (*r* = 0.31, *p* = 0.03).

**Fig. 3:**
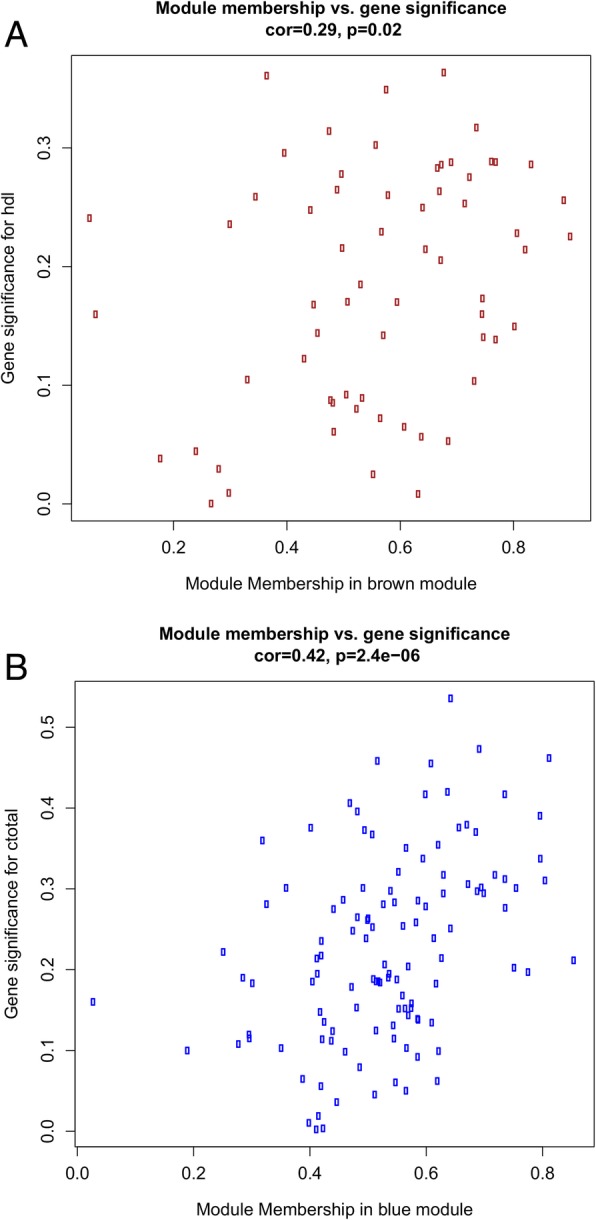
Scatterplots of gene significance for lipid profile traits and module membership in the **a** brown and **b** blue modules

Gene significance (GS), defined as the correlation between gene expression and lipid profile traits, was put in relation with module membership (MM), defined as the correlation of the ME and the gene expression profile. GS for HDL-C correlated with MM in the brown module (*r* = 0.29, *p* = 0.02), while GS for TC correlated with MM in the blue module (*r* = 0.42, *p* = 2.4 × 10^−6^) (Fig. 3). This suggests that genes highly significantly associated with HDL-C and TC were also the most important elements of the brown and blue modules, respectively. Thus, brown and blue modules were selected as modules of interest in subsequent analyses.

### Enrichment analysis

Enrichment analysis was performed for genes in brown and blue modules in order to elucidate biological mechanisms. In the GO database, a total of 73 GO terms from all three main categories (biological process, cellular component, and molecular function) were significantly enriched (corrected *p* ≤ 0.05) in the brown module (Additional file 3: Table S3). In the GO database, a total of 253 GO terms also from all three main categories were significantly enriched (corrected *p* ≤ 0.05) in the blue module (Additional file 4: Table S4). In the Reactome database, the most significant terms were used to better define modules of interest. Thus, the function representing the brown module was “apoptosis,” while the function representing the blue module was “mRNA splicing” (Additional file 3: Table S3 and Additional file 4: Table S4). Moreover, transcription factor (TF) enrichment analysis was conducted to identify TFs that are enriched in genes of the brown and blue modules. In the brown module, *NFE2L*2 was the more significantly enriched TF (*p* = 0.016) with target genes *MAF* and *MAFF*. In the blue module, *ESR1* was the most significantly enriched TF (*p* = 0.0047) with target genes *NOP56*, *ATF1*, *LDHA*, *SMARCB1*, *HNRNPA2B1*, *HNRNPR*, *SLC3A2*, *DNM1L*, *CCT7*, *HNRNPA1*, *DBN1*, and *MARK2*.

### Hub gene analysis

Hub gene analysis in brown and blue modules was conducted in order to refine the analysis of potential mechanisms. A total of seven transcripts (four hub genes: *CISH*, *FAM123C*, *ZSWIM4*, *FAM13A*) were identified in the brown module (Table 2). In the blue module, 32 transcripts (29 hub genes: *FEZ1*, *MYBPC3*, *OPRL1*, *SIRPB1*, *SLC16A3*, *SLC4A1*, *GPR89C*, *PSMD6*, *RANBP1*, *AP3M1*, *LOC221710*, *SIRT5*, *DNM1L*, *CR2*, *LBH*, *HNRNPA1*, *BIVM*, *NOP56*, *LOC728026*, *FBXO5*, *SNRPN*, *CCT7*, *TIAL1*, *SLC25A4*, *PCNA*, *PNN*, *S100PBP*, *UBE1DC1*, and *ZCCHC11*) were identified (Table 3). Classification terms (unknown, likely regulated, and likely regulator) were added in the Tables 2 and 3 to suggest potential causal relationships of the hub genes in the interconnection between total carotenoids and plasma lipid concentrations.

**Table 2:** Hub genes identified in the brown module

Probe ID	Gene (Chr, accession number)	GS HDL-C	*p* value GS	MM	*p* value MM	Potential causal relationships of the hub genes^1^
ID_4920064	*CISH* (Chr3, NM_145071)	0.29	0.049	0.67	1.44 × 10^−7^	Likely regulator
ID_6480731	*AMER3* (Chr2, NM_ 001105193)	0.36	0.011	0.68	1.12 × 10^−7^	Likely regulated
ID_3870603	*ZSWIM4* (Chr19, NM_ 023072)	0.29	0.047	0.69	5.12 × 10^−8^	Unknown
ID_290093	*LOC647322* (Chr2, withdrawn)	0.32	0.028	0.74	2.39 × 10^−9^	Unknown
ID_4540376	*FAM13A* (Chr4, NM_ 001015045)	0.29	0.047	0.76	2.88 × 10^−10^	Likely regulator
ID_2260315	*HS.572752*	0.29	0.047	0.77	1.48 × 10^−10^	Unknown
ID_3420706	*HS.566359*	0.29	0.049	0.83	2.03 × 10^−13^	Unknown

*Chr* chromosome, *GS* gene significance, *HDL-C* high-density lipoprotein cholesterol, *MM* module membership

^1^Classification terms have been added to suggest potential causal relationships of the hub genes in the interconnection between total carotenoids and plasma lipid concentrations

**Table 3:** Hub genes identified in the blue module

Probe ID	Gene (Chr, accession number)	GS TC	*p* value GS	MM	*p* value MM	Potential causal relationships of the hub genes^1^
ID_2350019	*FEZ1* (Chr11, NM_ 022549)	0.31	0.032	− 0.81	4.55 × 10^−12^	Unknown
ID_6940768	*MYBPC3* (Chr11, NM_000256)	0.34	0.019	− 0.80	1.06 × 10^−11^	Likely regulator
ID_1440754	*OPRL1* (Chr20, NM_000913)	0.30	0.037	− 0.70	3.76 × 10^−8^	Likely regulator
ID_5870341	*LOC642691* (Chr1, withdrawn)	0.30	0.040	− 0.69	5.85 × 10^−8^	Unknown
ID_3780017	*SIRPB1* (Chr20, NM_006065)	0.40	0.0049	− 0.68	1.01 × 10^−7^	Likely regulated
ID_110719	*SLC16A3* (Chr17, NM_004207)	0.30	0.037	− 0.62	2.45 × 10^−6^	Likely regulator
ID_2120152	*SLC4A1* (Chr17, NM_000342)	0.31	0.030	− 0.60	6.55 × 10^−6^	Likely regulated
ID_2630739	*HS.154948*	− 0.42	0.0032	0.60	6.37 × 10^−6^	Likely regulated
ID_6040072	*GPR89C* (Chr1, NM_001097616)	− 0.46	0.0012	0.61	4.10 × 10^−6^	Likely regulated
ID_5810070	*PSMD6* (Chr3, NM_014814)	− 0.36	0.012	0.62	3.19 × 10^−6^	Likely regulated
ID_7000735	*RANBP1* (Chr22, NM_002882)	− 0.38	0.0072	0.62	2.63 × 10^−6^	Likely regulated
ID_6200437	*AP3M1* (Chr10, NM_207012)	− 0.47	0.00076	0.62	2.35 × 10^−6^	Likely regulated
ID_4670703	*SMIM13* (Chr6, XM_927609)	− 0.35	0.013	0.62	2.29 × 10^−6^	Likely regulated
ID_1510270	*SIRT5* (Chr6, NM_012241)	− 0.29	0.042	0.63	1.48 × 10^−6^	Likely regulator
ID_3890632	*DNM1L* (Chr12, NM_012063)	− 0.32	0.028	0.63	1.46 × 10^−6^	Unknown
ID_4810292	*CR2* (Chr1, NM_001877)	− 0.42	0.0030	0.64	1.03 × 10^−6^	Likely regulated
ID_2810246	*LBH* (Chr2, NM_030915)	− 0.46	0.0011	0.64	9.22 × 10^−7^	Likely regulated
ID_10209	*HNRNPA1* (Chr12, NM_031157)	− 0.53	8.78 × 10^−5^	0.64	7.83 × 10^−7^	Likely regulator
ID_1090367	*BIVM* (Chr13, NM_017693)	− 0.38	0.0085	0.66	3.64 × 10^−7^	Likely regulated
ID_5560465	*NOP56* (Chr20, NM_006392)	− 0.38	0.0079	0.67	1.70 × 10^−7^	Likely regulated
ID_1990154	*PTMAP12* (Chr9, XM_001126659)	− 0.31	0.035	0.67	1.51 × 10^−7^	Unknown
ID_4010097	*FBXO5* (Chr6, NM_012177)	− 0.42	0.0027	0.68	1.37 × 10^−7^	Likely regulated
ID_7100367	*HS.571502*	− 0.39	0.0057	0.69	7.78 × 10^−8^	Likely regulated
ID_4850133	*SNRPN* (Chr15, NM_022806)	− 0.37	0.0096	0.69	6.75 × 10^−8^	Likely regulated
ID_7160719	*CCT7* (Chr2, NM_006429)	− 0.47	0.00069	0.69	4.71 × 10^−8^	Likely regulated
ID_5560504	*TIAL1* (Chr10, NM_003252)	− 0.29	0.042	0.70	2.85 × 10^−8^	Unknown
ID_6840747	*SLC25A4* (Chr4, NM_001151)	− 0.32	0.028	0.72	7.82 × 10^−9^	Unknown
ID_6900079	*PCNA* (Chr20, NM_182649)	− 0.42	0.0032	0.74	2.27 × 10^−9^	Likely regulated
ID_7150132	*PNN* (Chr14, NM_002687)	− 0.31	0.031	0.74	2.23 × 10^−9^	Unknown
ID_3170189	*S100PBP* (Chr1, NM_022753)	− 0.30	0.038	0.76	4.91 × 10^−10^	Unknown
ID_110059	*UBA5* (Chr3, NM_024818)	− 0.39	0.0061	0.80	1.12 × 10^−11^	Likely regulated
ID_4260093	*TUT4* (Chr1, NM_001009882)	− 0.46	0.00096	0.81	2.10 × 10^−12^	Likely regulated

*Chr* chromosome, *GS* gene significance, *MM* module membership, *TC* total cholesterol

^1^Classification terms have been added to suggest potential causal relationships of the hub genes in the interconnection between total carotenoids and plasma lipid concentrations

## Discussion

We first tested the association between whole blood genome-wide gene expression levels and plasma total carotenoid concentrations in order to identify probes influenced by carotenoids. A total of 533 probes were significantly associated with total carotenoids. To the best of our knowledge, this is the first study that considers the impact of plasma total carotenoids on genome-wide gene expression levels. Up to now, studies focused mainly on the effect of genetic variants on the inter-individual variability in β-carotene, lycopene, and lutein bioavailability in response to dietary interventions [22–24]. SNPs were located in genes mainly related to fatty acids and cholesterol absorption including *SR-B1*, *CD36*, *NPC1L1*, and *ABCA1.* Since several proteins are involved in the absorption and metabolism of carotenoids, several papers have suggested that variations in the expression of the genes encoding for these proteins might also impact carotenoid bioavailability [25]. Moreover, carotenoids modulate the mechanisms of cell proliferation, growth factor signaling, and gap junction communication, and lead to changes in the expression of many proteins involved in these processes [20]. The effect of carotenoids on gene expression may result from direct interaction with ligand-activated nuclear receptors such as retinoid receptors, the nuclear factor-kappa B, and the peroxisome proliferator-activated receptors [20, 26, 27]. In accordance, two important TFs were enriched among genes associated with total carotenoids. *NFE2L2*, which encodes a TF regulating genes with antioxidant response elements, was enriched in the brown module. Interestingly, some carotenoids have been showed to protect against light-induced cell damage through *NFE2L2* activation [28]. *ESR1*, a ligand-activated TF, was enriched in the blue module. Genetic variations in this gene were associated with serum TC and LDL-C concentrations [29].

Second, WGCNA was used to identify modules and key genes related to lipid profile in the subset of 533 probes associated with carotenoids. MEs of the turquoise and blue modules had a significant correlation with HDL-C and TC, while ME of the brown module had a significant correlation with HDL-C. This suggests that highly co-expressed genes in these modules had potential interaction or consistent biological effects on HDL-C and TC. Only the brown and the blue modules showed a significant correlation between the GS for HDL-C and TC, respectively, and the MM of the module. Functional enrichment analysis revealed several enrichments of categories related to cell metabolism and processes. However, functional enrichment analysis yields large amount of GO terms and Reactome terms non-specific to lipid profile. Thus, it was not precise enough to identify specific pathways or genes related to lipid or carotenoid metabolism.

The analysis with hub genes allowed refining the analysis of potential mechanisms linking carotenoids and lipid profile. A total of four hub genes were identified in the brown module: *FAM123C*, *ZSWIM4*, *FAM13A*, and *CISH. FAM13A* and *CISH* are of interest in the context of lipid profile. *FAM13A* encodes for a family with sequence similarity 13 member A. *FAM13A* variants have been associated with HDL-C in individuals from various descents [30, 31]. *FAM13A* promoted fatty acid oxidation, possibly by interacting with an activating sirtuin 1 and increasing expression of *CPT1A* [32]. Interestingly, another study using WGCNA identified *FAM13A* as a hub gene related to hyperlipidemia [33]. *CISH* encodes for a cytokine-inducible SH2 containing protein. It controls the signaling of a variety of cytokines, in particular interleukin-2, and seems to be critical for T cell proliferation and survival in response to infection [34]. Despite the fact that expression of *CISH* was not inhibited in the presence of delipidated HDL lipoproteins in a cell study, the link is plausible since HDL specifically inhibits the production of pro-inflammatory cytokines, which is also controlled by CISH [35]. A total of 29 hub genes were identified in the blue module. Relevant genes related to lipid and carotenoid metabolism include *MYBPC3*, *OPRL1*, *SLC16A3*, *SIRT5*, *HNRNPA1*, *SLC25A4*, and *PCNA*. *MYBPC3* encodes for a myosin binding protein C, cardiac. Mutation in this gene (*MYBPC3-*Q1061X) results in hypertrophic cardiomyopathy and cardiac oxidative stress with elevated TG and branched-chain amino acid levels [36, 37]. *OPRL1* encodes for an opioid-related nociceptin receptor 1 involved in many biological functions including stress, inflammatory, and immune responses. A study by our group identified interaction effects between 29 SNPs, including rs2229205 in *OPRL1*, and total fat intake on LDL peak particle diameter [38]. *SLC16A3* encodes for a proton-linked monocarboxylate transporter designated solute carrier family 16 member 3. Monocarboxylate transporters are involved in the transport of short-chain fatty acids and may also be involved in the transport of cholesterol-lowering agents [39]. *SLC16A3* was significantly upregulated in 102 men receiving an antioxidant-rich diet compared to controls [40]. *SIRT5* encodes for sirtuin 5, which is involved in the regulation of mitochondrial metabolism, oncogenesis, and oxidative stress [41, 42]. Obese Sirt5−/− mice showed increased serum cholesterol concentrations compared to Sirt5+/+ mice [43]. *HNRNPA1* encodes for a heterogeneous nuclear ribonucleoprotein A1, which has been shown to reduce HMGCR enzyme activity and increase LDL-C uptake [44]. *SLC25A4* encodes for a solute carrier family 25 member 4. A variation in this gene is associated with hypertrophic cardiomyopathy [45]. Linoleic acid also increased *ANT1* (*SLC25A1*) expression as a compensatory response to an increase in intracellular ROS [46]. Finally, *PCNA* encodes for a proliferating cell nuclear antigen, which has high expression in tumor tissues [47]. Interestingly, carotenoids present various suppressive abilities against *PCNA* expressions in cell proliferation [48]. Lycopene also decreased the positive rate of PCNA and protein expressions of PCNA in lung tissue [49]. In summary, several hub genes were related to lipid metabolism, oxidative stress, or antioxidant action of carotenoids. Thus, the plausible link between carotenoids and lipid profile does not seem to be entirely due to the fact that carotenoids are transported by lipoproteins. Indeed, circulating carotenoids have been showed to impact TFs involved in lipid metabolism [50, 51]. Accordingly, in the present study, plasma carotenoids modulated expression of several genes related, among others, to lipid metabolism. Moreover, classification terms were used to suggest potential causal relationships of the hub genes in the interconnection between total carotenoids and plasma lipid concentrations. A total of seven transcripts were classified as “likely regulator” as they have been associated with or shown to influence lipid levels in the literature and thus represent potential regulators of lipid concentrations. For example, *FAM13A*, which is associated with total carotenoids, also demonstrated a regulatory effect on HDL-C via its genetic variations and effect on fatty acid oxidation [30–32]. A total of 20 transcripts were classified as “likely regulated” meaning that they were associated with both total carotenoids and lipid concentrations in the present study. Finally, a total of 12 transcripts were classified as “unknown” considering they showed only associations with total carotenoids.

The present study has strengths but also some limitations. The main strength results from the study of the combination of both genome-wide gene expression levels and total carotenoids, representing six predominant plasma carotenoids. To the best of our knowledge, this is the first study that considers the impact of plasma total carotenoids on genome-wide expression levels. Another strength is the use of WGCNA that adds important information about the effect of co-expression network of genes, which is useful to detect biological pathways related to lipid profile. On the other hand, the study’s main limitation resides in the small sample size, which limits statistical power. However, the study of a founder population with relatively homogeneous genetics and shared environment is a new aspect in this field [52]. Nonetheless, this limits the generalization of results to other populations. Finally, our study did not account for diet, physical activity, smoking, and alcohol consumption of participants, all of which may affect circulating carotenoid concentrations [53, 54].

## Conclusions

In conclusion, whole blood expression levels of 533 probes were associated with plasma total carotenoid concentrations. Using WGCNA, four modules were identified. A total of four and 29 hub genes associated with total carotenoids were potentially related to HDL-C and TC, respectively. This integrative analysis provides evidence for the potential role of gene co-expression in the relationship between carotenoids and lipid concentrations. Further studies and validation of the hub genes are needed. Finally, this article may also serve as an example of how to include a wide range of omics data in nutrition studies, using systems biology methods.

## Methods

### Patients and design

A total of 48 Caucasian French-Canadian subjects from 16 families were recruited in the greater Quebec City metropolitan area, in Canada, as part of the GENERATION Study. The GENERATION Study was designed to evaluate familial resemblances in omics (DNA methylation [55] and gene expression [56]) and metabolic (metabolites [57] and carotenoids [7]) profiles in healthy families and to test the impact of these profiles on cardiometabolic health. Families were composed of 16 mothers, 6 fathers, and 26 children. Families living under the same roof comprised at least the mother and one child aged between 8 and 18. Parents had to be the biological parents of their child (or children), in good general health, non-smokers, with body mass index (BMI) ranging between 18 and 35 kg/m^2^, and free of any metabolic conditions requiring treatment, although the use of Synthroid® (levothyroxine) or oral contraceptive was tolerated. Children also had to be non-smokers, in good general health, and not using psycho-stimulators [Ritalin® (methylphenidate), Concerta® (methylphenidate), and Strattera® (atomoxetine)]. Blood samples were taken from both parents and children during their visit at the Institute of Nutrition and Functional Foods (INAF). The experimental protocol was approved by the Ethics Committees of Laval University Hospital Research Center and Laval University. All participants (adults and children) signed an informed consent document. Parental consent was also obtained by signing the child consent document.

### Anthropometric and cardiometabolic measurements

Body weight, waist girth, and height were measured according to the procedures recommended by the Airlie Conference [58]. Blood samples were collected from an antecubital vein into vacutainer tubes containing EDTA after 12-h overnight fast and 48-h alcohol abstinence. Plasma was separated by centrifugation (2500 g for 10 min at 4 °C), and samples were aliquoted and frozen (− 80 °C) for subsequent analyses. Enzymatic assays were used to measure plasma TC and TG concentrations [59, 60]. Precipitation of very-low-density lipoprotein (VLDL) and LDL particles in the infranatant with heparin-manganese chloride generated the HDL-C fraction [61]. LDL-C was calculated with the Friedewald formula [62]. ApoB100 concentrations were measured in plasma by the rocket immunoelectrophoretic method [63]. Using a sensitive assay, plasma C-reactive protein (CRP) was measured by nephelometry (Prospec equipment Behring) [64].

### RNA extraction and gene expression analysis

Total RNA was isolated and purified from whole blood using PAXgene Blood RNA Kit (Qiagen). Quantification and verification of the purified RNA was assessed using both the NanoDrop (Thermo Scientific, Wilmington, DE, USA) and the 2100 Bioanalyzer (Agilent Technologies, Cedar Creek, TX, USA). The HumanHT-12 v4 Expression BeadChip (Illumina Inc., San Diego, CA) was used to measure expression levels of ~ 47,000 probes (> 31,000 annotated genes). This was performed at the McGill University and Genome Quebec Innovation Centre (Montreal, Canada). The FlexArray software (version 1.6) [65] and the lumi R package were used to analyze and normalize gene expression levels. Probes with a detection *p* value ≤ 0.05 in at least 25% of all subjects were considered in the analysis. A total of 18,160 probes among the 47,323 probes on the microarray (38.4%) showed significant gene expression in the blood.

### Plasma carotenoid measurements

Samples and standards used for the measurement of carotenoid concentrations were prepared as reported previously [7]. Briefly, 100 μL of fasting plasma samples were thawed a few hours before analysis. Plasma samples were transferred to Eppendorf tubes with 20 μL of 2-propanol and 20 μL of carotenoid standard. Samples were transferred on a 400-μL fixed well plate (ISOLUTE® SLE+, Biotage, Charlotte, NC) with 900 μL of hexane to isopropanol (90/10, *v*/*v*) in each well. Each extracted sample was evaporated under nitrogen and reconstituted with 300 μL of methanol to dichloromethane (65/35, *v*/*v*). Plates were shaken for 10 min and samples were transferred into high-performance liquid chromatography glass vials for analysis.

High-performance liquid chromatography (HPLC)-UV analysis was performed using an Agilent 1260 liquid handling system (Agilent, Mississauga, Ontario, Canada) as described previously [7]. Carotenoids were separated with a mobile phase consisting of methanol to water (98/2, *v*/*v*; Eluent A) and methyl-tert-butyl ether (MTBE; Eluent B; VWR, Mississauga, Ontario, Canada). Flow-rate was set at 1 mL/min and the gradient elution was as follows: 2% Eluent B (initial), 2.0–80% Eluent B (0.0–27.0 min), isocratic 80% Eluent B (27.0–31.0 min), 80.0–2.0% Eluent B (31.0–31.1 min), and isocratic 2% Eluent B (31.1–34.0 min). UV detector was set at 450 nm, and identification of each compound was confirmed using retention time and UV spectra (190–640 nm) of the pure compounds. Data acquisition was carried out with the Chemstation software (Agilent, Mississauga, Ontario, Canada). For all carotenoids, the concentrations are reported in micromoles per liter of plasma. One outlier in β-crypoxanthin, defined as value falling outside of the mean ± 4 standard deviations, was excluded from analyses.

### Association between total carotenoids and gene expression levels

Total plasma carotenoid (micromoles per liter of plasma) concentrations were calculated as the sum of α-carotene, β-carotene, β-cryptoxanthin, lutein, lycopene, and zeaxanthin concentrations. Concentrations of plasma carotenoids are available in Additional file 1: Table S1. R software v2.14.1 (R Foundation for Statistical Computing; http://www.r-project.org) [66] was used to compute regressions between normalized gene expression levels of all 18,160 probes and total carotenoids adjusted for the family ID. Weighted gene co-expression network analysis (WGCNA) was performed with gene expression levels of 533 probes showing a significant association (*p* value ≤ 0.05, obtained from the linear model function) with total carotenoids.

### Weighted gene co-expression network analysis (WGCNA)

WGCNA was performed with the WGCNA package [21, 67] in R software [66]. First, co-expression was measured between each gene pair using Pearson correlation coefficients (varying from − 1 to 1). Considering the small sample size of the present study, Pearson correlations measuring linear relationships were selected to avoid the pitfall of overfitting [68]. In order to transform the correlation coefficients into a weighted adjacency matrix (values ranging from 0 to 1), the co-expression similarity was raised to a power *β* = 6 [68]. The adjacency matrix allows measuring the connection strengths between nodes. From this matrix, we can build the topological overlap matrix (TOM) that considers the topological similarity. The TOM was then used to calculate the corresponding dissimilarity (1-TOM) in order to form clusters. Average linkage hierarchical clustering coupled with the TOM-based dissimilarity was used to group genes with coherent expression profiles into modules [68]. More specifically, the dynamic tree cutting algorithm (deep split = 2, minimum number of genes per modules = 30, cut height = 0.25) was used to detect gene modules (clusters of densely interconnected genes in terms of co-expression). The Partitioning Around Medoids (PAM) method was also used to allow the assignment of outlying genes to modules. Colors are randomly assigned to modules except for the gray color, which is reserved for the module with unassigned genes. To identify modules that were significantly associated with lipid profile traits (TC, LDL-C, HDL-C, TG, ApoB100), correlations between module eigengenes (MEs) (i.e., the first principle component of the module, which represents the overall expression level of the module) [69] and traits were computed. Gene significance (GS), defined as the absolute correlation between the gene and the trait, was used to quantify associations of individual genes with lipid profile traits. To quantify the similarity of all genes to every module, a quantitative measure of module membership (MM) was defined as the correlation of the ME and the gene expression profile. Genes with the highest MM and highest GS were those with high significance (hub genes) [70]. The hub genes within a module were chosen based on GS > 0.2 and MM > 0.6, with a *p* value ≤ 0.05. All these analyses were computed using commands implemented in the WGCNA package.

### Functional enrichment analysis and hub genes classification

Reactome and Gene Ontology (GO) enrichment analysis was conducted with KEGG Orthology-Based Annotation System (KOBAS) on the genes of modules of interest [71]. Hypergeometric test/Fisher’s exact test was used to identify significant pathways. FDR correction method (Benjamini and Hochberg) was used to account for multiple testing in enrichment analysis. The most significant Reactome terms were used to characterize brown and blue modules. TFs enrichment analysis was conducted using Enrichr (http://amp.pharm.mssm.edu/Enrichr/), an online biological information database that integrates several biological databases [72]. The most significant TFs in each module were retained. Moreover, analyses were carried out in order to suggest potential implications of hub genes in the relationship between carotenoids and lipids. First of all, regressions between expression levels of all hub genes and plasma lipid concentrations adjusted for the family ID were computed in Statistical Analysis Software (SAS) version 9.4. More specifically, the seven transcripts of the brown module were tested for associations with HDL-C and the 32 transcripts of the blue module were tested for associations with TC. Significant associations (*p* ≤ 0.05) are presented in Additional file 5: Table S5. Considering that all hub genes initially showed associations with total carotenoids, potential causal relationships of hub genes were classified as follows: (1) unknown: genes that showed association with carotenoids but not with lipids, (2) likely regulated: genes that showed association with both carotenoids and lipids, and (3) likely regulator: genes that showed association with carotenoids and either have been associated with lipids in literature or showed association with lipids in addition to being shown to influence lipid levels in the literature. The literature of associations of genes with lipids is detailed in the discussion. These classification terms were used to quickly have an idea of the potential implications of hub genes in the relationship between carotenoids and lipids.

### Statistical analysis

SAS version 9.4 was used to compute sex differences in cardiometabolic parameters between fathers and mothers and between daughters and sons using an unpaired *t* test.

## Additional files

Additional file 1: **Table S1.** Concentrations of plasma carotenoids (micromoles per liter of plasma). (XLSX 15 kb)

Additional file 2: **Table S2.** Probes showing significant association with plasma total carotenoids (*n* = 533) (XLSX 42 kb)

Additional file 3: **Table S3.** Functional enrichment analysis in the brown module using GO and Reactome databases (XLSX 15 kb)

Additional file 4: **Table S4.** Functional enrichment analysis in blue module using Gene Ontology and Reactome databases. (XLSX 28 kb)

Additional file 5: **Table S5.** Significant *p* values of regressions between expression levels of hub genes and plasma lipid concentrations. (XLSX 11 kb)

## Abbreviation

ApoB100: Apolipoprotein B100
GO: Gene Ontology
GS: Gene significance
HDL-C: High-density lipoprotein cholesterol
LDL-C: Low-density lipoprotein cholesterol
ME: Module eigengenes
MM: Module membership
SD: Standard deviation
TC: Total cholesterol
TF: Transcription factor
TG: Triglycerides
TOM: Topological overlap matrix
WGCNA: Weighted gene co-expression network analysis
